# Cyclosporine eye drops in lattice corneal dystrophy type 1: case report

**DOI:** 10.1097/MS9.0000000000003625

**Published:** 2025-07-22

**Authors:** Hasan al-Hawasli, Raghid Tarbouche

**Affiliations:** a Scientific Affairs Deputy Manager, Oubari Habboush Pharma, Damascus, Syria; b Eye and Ear Hospital, Damascus, Syria

**Keywords:** bilateral, case report, cyclosporine, lattice corneal dystrophy

## Abstract

**Introduction and importance:**

To report a successful treatment of lattice corneal dystrophy type 1 (LCD1) using topical cyclosporine (CsA) eye drop emulsion 0.05%.

**Case presentation:**

A 48-year-old female patient presented with bilateral LCD1characterized by corneal stromal lattice lines and visual symptoms. The patient was treated with topical CsA 0.05% twice daily for 3 months, followed by once daily for another 3 months.

**Clinical discussion:**

Following treatment, the patient experienced a significant improvement in symptoms and resolution of corneal opacities in the treated eye.

**Conclusion:**

This case report demonstrates the potential efficacy of topical CsA as a novel treatment option for LCD1, offering a noninvasive approach to manage symptoms and improve visual function. Further studies are needed to confirm these findings.

## Introduction

Lattice corneal dystrophy (LCD) is an inherited disorder of the eye characterized by the bilateral deposition of amyloid resulting in steadily progressive loss of vision. These deposits create linear, “lattice-like” opacities arising primarily in the central cornea, while the peripheral cornea is often spared. It can cause a variety of symptoms including blurry vision, glare, and pain, significantly impacting daily life. While there is no cure for LCD, current treatment options aim to manage symptoms^[1]^. Approximately 60% of all corneal dystrophies are endothelial, whereas such conditions as macular, lattice, and granular corneal dystrophies are far less prevalent, each making up 1% or less of the total^[2]^.

Type I LCD (LCD1), also known as classic LCD or Biber–Haab–Dimmer dystrophy, is the primary form of LCD. It was first described in 1890 by Hugo Biber^[3]^. It is autosomal dominant and results from mutations in the transforming human growth factor beta-induced (*TGFBI*) gene. Although TGFBI and its protein transcript are found throughout the body, there are no known systemic effects outside of the ocular pathology for which it is named. It usually presents in the first or second decade of life^[4]^.

**HIGHLIGHTS**

- This case report highlights a successful treatment of lattice corneal dystrophy type 1 (LCD1) using topical cyclosporine (CsA) eye drops.
- A 48-year-old female patient with LCD1 experienced significant symptom improvement and resolution of corneal opacities in one eye after topical CsA treatment.
- CsA may offer a novel, noninvasive treatment option for LCD1.

Topical cyclosporine (CsA) has been shown to be beneficial in patients with familial amyloid polyneuropathy after liver transplant, showing symptomatic improvement and improving the quality of life^[5]^. CsA reduces inflammation and improves the composition of the tear film through its inhibitory action on T lymphocytes and by increasing goblet cells in the conjunctival epithelium. Its topical use is not associated with adverse effects and it has a low systemic absorption^[6]^. This case demonstrates the potential of CsA eye drops as a new treatment option for LCD1. Compared to other interventions like penetrating keratoplasty (PK) and deep anterior lamellar keratoplasty, CsA offers a potentially safer and less invasive approach. This case report has been reported in line with the SCARE 2025 Criteria^[7]^.

## Case report

A 48-year-old Caucasian woman presented (12 December 2023) with LCD1 in both eyes. She had experienced symptoms (blurry vision, glare) over the past 15 years, but her daily activities were not significantly impacted. The patient had no previous medical or laser treatments.

**Figure 1.**
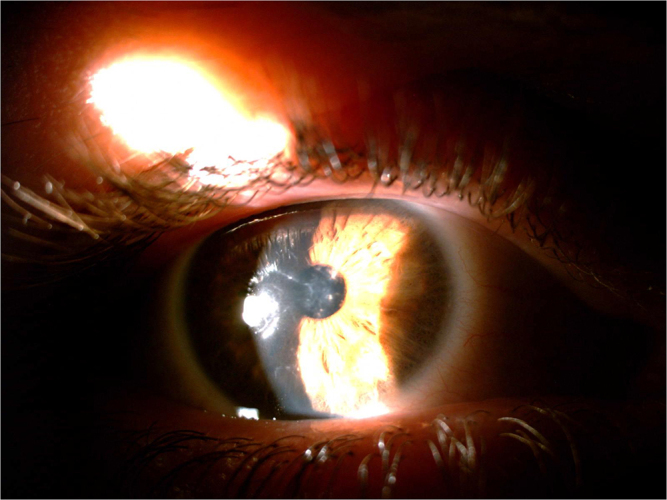
Photograph of the right eye (the corneal stroma shows lattice-like stripe turbidity) as shown by slit-lamp examination.

**Figure 2.**
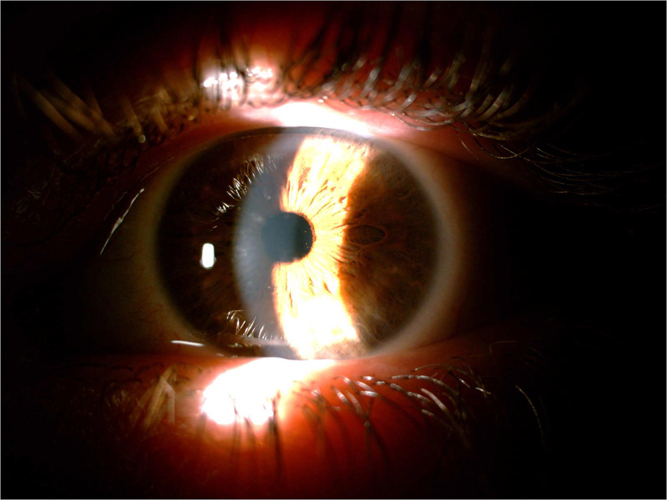
Photograph of the left eye (the corneal stroma shows lattice-like stripe turbidity) as shown by slit-lamp examination.

Clinical symptoms and slit-lamp examination findings confirmed the diagnosis of LCD1. Amyloid deposits were found throughout the corneal stroma and this coincides with the pattern of lines typical of this lattice dystrophy (Figures 1 and 2). Visual acuity testing revealed a visual acuity of 3/10 in the right eye (RE) and 8/10 in the left eye (LE). Intraocular pressure was 16 mmHg in both eyes and fundus observation showed no alterations. There was a familial history of corneal dystrophy (i.e., the patient’s mother and brother both eyes).

Her primary complaint at her visit was dry eye and pain in her RE (severe dry eye disease, ocular surface disease index > 33), so she was treated with CsA eye drops (Lacrosporin; ophthalmic emulsion 0.05%; Sina Darou Laboratories Co.) in the RE twice daily for 3 months, followed by once daily for another 3 months. Prednisolone eye drops were occasionally used to manage inflammation attacks. This treatment plan was not applied to her LE.

**Figure 3.**
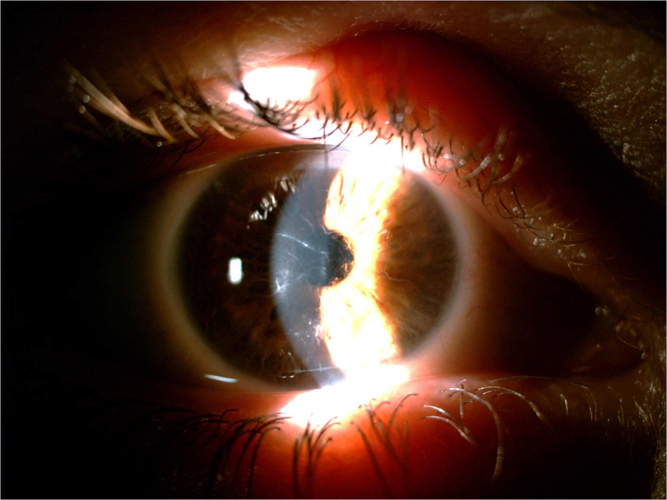
Slit-lamp photographs of the patient’s right eye after treatment.

**Figure 4.**
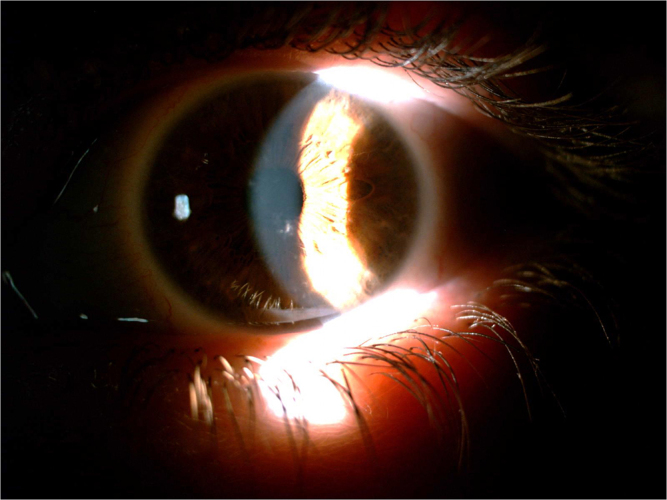
Slit-lamp photographs of the patient’s left eye after treatment of the right eye.

Following about 6 months of treatment (27 May 2024), the patient experienced an improvement in dry eye symptoms, going from severe to moderate to mild in her RE, without any side effects. Additionally, slit-lamp examination findings showed a good resolution (almost disappeared) of the corneal changes (characterized by the evanescence of amyloid “lattice-like”) (Figure 3). This improvement in symptoms and corneal health was maintained for three months following the end of treatment in the RE. As a control; the LE deposition of amyloid “lattice-like” was not changed after 6 or 9 months from her first visit (Figure 4).

## Discussion

This case report describes the successful use of CsA eye drops (ophthalmic emulsion 0.05%) in the RE of a patient with LCD1 – with the LE as a control – highlighting its potential as a new treatment approach.

After observing the patient’s case, and to establish the novelty of this case report, a comprehensive literature review was conducted using the following keywords and search terms across PubMed, and Google Scholar: “Lattice Corneal Dystrophy Type 1,” “LCD1,” “Cyclosporine,” “Cyclosporine eye drops,” “Topical cyclosporine,” “Corneal dystrophy treatment,” “Non-surgical LCD1 treatment,” and “Case report LCD1 cyclosporine,” including combinations of these terms. The search focused on identifying similar case reports, clinical trials, or studies that reported the use of topical CsA for the treatment of LCD1. While studies on CsA use for other corneal conditions and the Kayukawa et al. case report on systemic CsA in a patient with Mooren’s ulcer and LCD1 were found^[8]^, no similar reports specifically detailing the successful use of topical CsA eye drops as a primary treatment for LCD1 were identified.

LCD1 is characterized histopathologically by multiple eosinophilic amyloid inclusions between the epithelium and the Bowman layer, as well as within the corneal stroma. The deposits stain well with Congo red, exhibiting birefringence and dichroism under polarized light. They also show metachromasia when stained with crystal violet, and fluorescence when stained with thioflavin T^[1]^.

In LCD1 patients, phototherapeutic keratectomy (PTK) is sometimes performed because of a significant anterior surface lesion. The best LCD1 treatment is surgical procedures, such as PTK and deep anterior lamellar keratoplasty, which are relatively limited to the anterior surface, considering only the astigmatism perspective^[9]^.

Kayukawa et al^[8]^. described a successful use of systemic CsA in a patient with bilateral Mooren’s ulcer occurring in a case of LCD1 (62-year-old man) (the rationale of cyclosporin is for the immunomodulation of Mooren’s ulcer but not LCD1). The findings in that case indicate that it is possible to perform aggressive surgical treatments such as cataract surgery and PK in cases of Mooren’s ulcer when the disease is under appropriate management with systemic CsA. Both our case and the case discussed observed improvements in symptoms and visual acuity after a long duration of symptoms (about 15–20 years). However, there are some key differences. In our case, the patient used a topical treatment course (6 months) to achieve the resolution in her RE. The use of topical CsA eye drops presents a non-surgical alternative for managing LCD1. Unlike invasive procedures such as PTK or keratoplasty, which carry risks of surgical complications like infection, graft rejection, and scarring, CsA offers a less invasive approach^[10]^.

CsA is an immunosuppressant medication that modulates the activity of T cells^[11]^. CsA inhibits IL-13 synthesis^[12]^. CsA can also affect mitochondrial activity in some cells. In human conjunctival epithelial cells, the inflammatory mediators tumor necrosis factor-alpha and interferon-gamma induce mitochondrial permeability transition pore opening, upregulate Fas/FasL and caspase, and increase cell apoptosis. CsA prevents epithelial cell death^[13]^.

Recently, IL-13 was shown to be an important contributor to conjunctival fibrosis and inflammation in Ocular Cicatricial Pemphigoid. IL-13 stimulates collagen lattice contraction and migration, and mutes MMP3 and 10 production. The investigators speculated that in this setting fibroblasts provide an additional link to autoreactive T-cell activation by assuming the role as antigen-presenting cells, as IL-13 also augmented expression of molecules required for T-cell costimulation (CD80, CD40), and antigen presentation (MHC class II) on fibroblasts^[14]^.

While not an established treatment for LCD1, its immunomodulatory properties may be beneficial in managing the underlying immune response thought to contribute to the disease symptoms.

Further studies with larger patient cohorts are necessary to understand the optimal treatment regimen for CsA in LCD1.

A limitation of this case report is the absence of precise numerical data for the improvement in visual acuity and corneal opacity resolution following treatment with topical CsA. While baseline visual acuity was documented as 3/10 in the RE and 8/10 in the LE, incomplete record-keeping prevented us from obtaining quantitative measurements of visual acuity and corneal opacity at follow-up. Instead, we relied on qualitative observations, noting an improvement in dry eye symptoms from severe to mild in the RE and a “good resolution (almost disappear)” of the corneal changes based on slit-lamp examination. This lack of numerical data limits the ability to objectively quantify the treatment’s effectiveness on these specific parameters. Also, corneal biopsy and histopathological examination were not performed in this case. This was primarily due to limitations in the availability of specialized facilities and resources required for processing and analyzing corneal tissue samples in a timely manner.

## Conclusion

To the best of our knowledge, this is the first case report that presents the successful use of CsA eye drops (ophthalmic emulsion 0.05%) in treating LCD1. The patient experienced a significant improvement in symptoms and corneal health in the treated eye. Further research is needed to confirm the efficacy and safety of CsA for LCD1. However, this case suggests a new area of exploration for treatment options in this condition.

## Data Availability

Not applicable.
